# Supplementary material for: Oral Capecitabine-Vinorelbine Is Associated with Longer Overall Survival When Compared to Single-Agent Capecitabine in Patients with Hormone Receptor-Positive Advanced Breast Cancer
Source: Cancers (Basel). 2020 Mar 6;12(3):617. doi: 10.3390/cancers12030617 (PMC7139362; doi:10.3390/cancers12030617)
Supplement: Supplementary file 1 [file cancers-12-00617-s001.pdf]

Supplementary Materials

Oral Capecitabine-Vinorelbine is Associated with Longer Overall Survival When Compared to Single-Agent Capecitabine in Patients with Hormone Receptor-Positive Advanced Breast Cancer

Claudio Vernieri, Michele Prisciandaro, Federico Nichetti, Riccardo Lobefaro, Giorgia Peverelli, Francesca Ligorio, Emma Zattarin, Maria Silvia Cona, Pierangela Sepe, Francesca Corti, Sara Manglaviti, Marta Brambilla, Barbara Re, Antonino Belfiore, Giancarlo Pruneri, Luigi Celio, Gabriella Mariani, Giulia Valeria Bianchi, Licia Rivoltini, Giuseppe Capri and Filippo de Braud

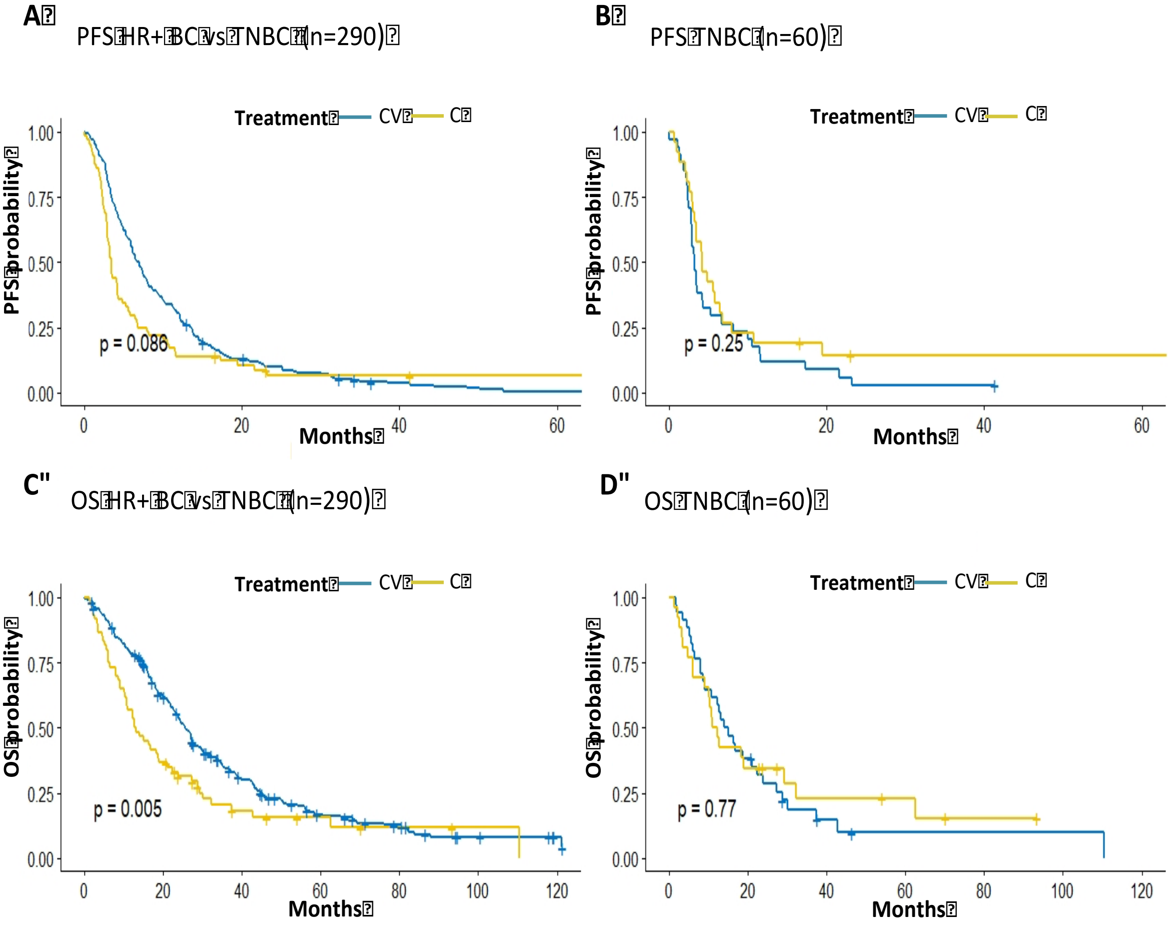

Figure S1. A: Kaplan Meier curves of PFS in the patients with HR+ BC vs. TNBC regardless of the treatment cohort. **B:** Kaplan Meier curves of PFS in TNBC patients treated with CV vs. C. **C:** Kaplan Meier curves of OS in the patients with HR+ BC vs. TNBC regardless of the treatment cohort. **D**: Kaplan Meier curves of PFS in TNBC patients treated with CV vs. C. The + symbol indicates patients censored at the time of data cut off and analysis. For each comparison, the *p* value of the Log-rank test is indicated.

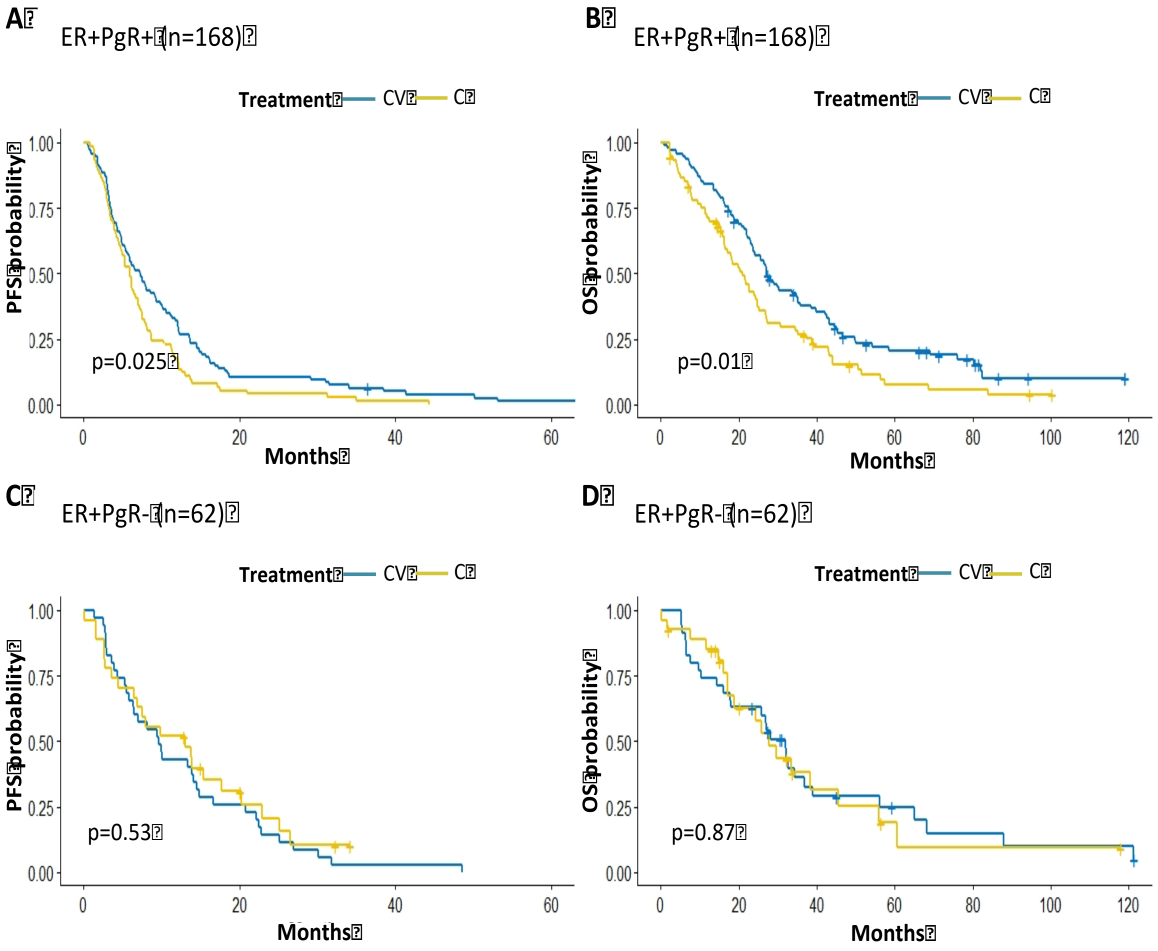

Figure S2. A,B: Kaplan Meier curves of PFS (**A**) and OS (**B**) in the subgroup of patients with ER+ PgR+ BC; **C,D:** Kaplan Meier curves of PFS (**C**) and OS (**D**) in the subgroup of patients with ER+ PgR- BC. For each indicated comparison, the *p* value of the Log-rank test is indicated.

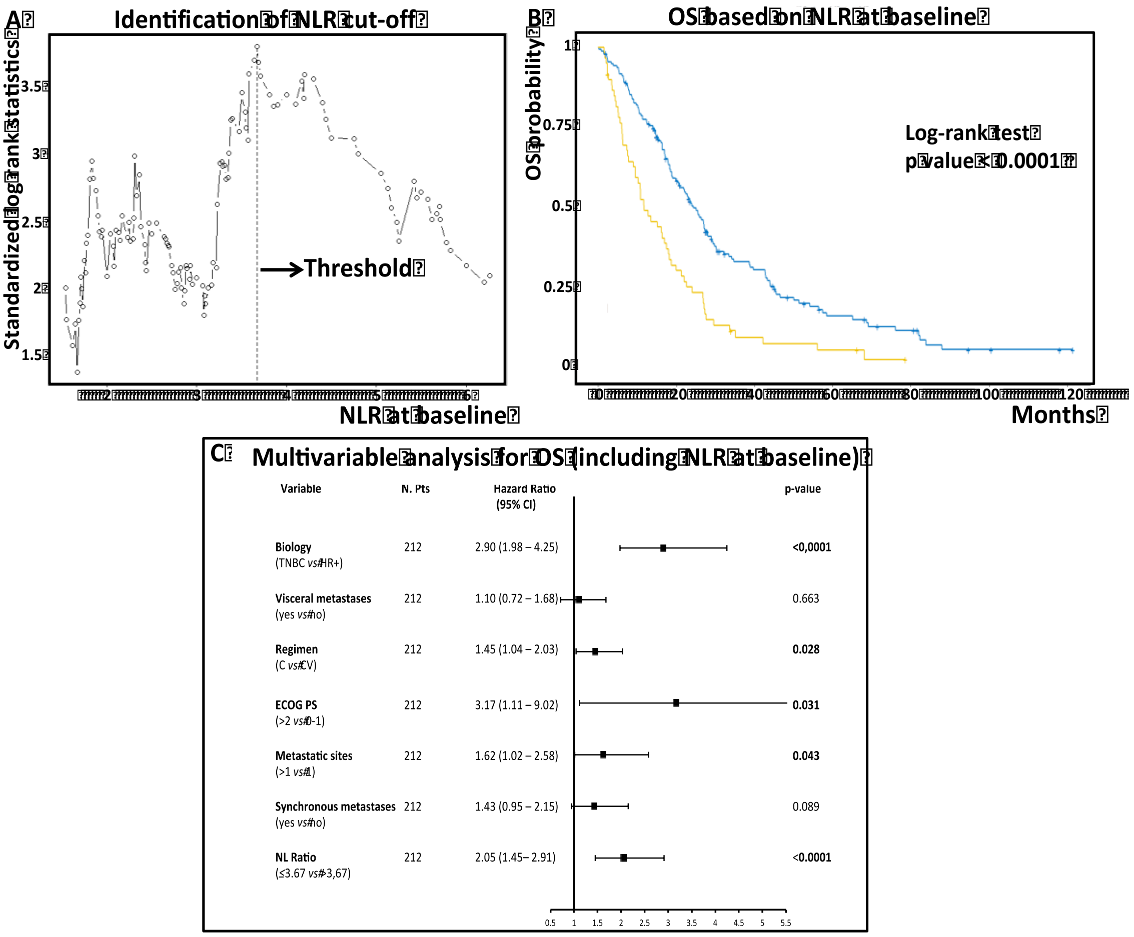

Figure S3. Identification of the best baseline NLR threshold to discriminate patients on the basis of their OS (**A**). Kaplan Meier OS curves in patients with baseline NLR lower (blue curve) or higher (yellow curve) than the previously identified threshold (**B**). Forest plots indicating the adjusted hazard ratios and 95% CIs for OS of clinical and tumor-related variables, including baseline NLR (**C**).

Table S1. Clinical and Tumor Characteristics in patients matched on the basis of covariates independently associated with the risk of death.

Characteristic	Overall n = 238	C subgroup n = 119	CV subgroup n = 119	p
**Age, Years**				
≥65	120 (50.4)	80 (67.2)	40 (33.6)	
<65	118 (49.6)	39 (32.8)	79 (66.4)	**<0.01**
**ECOG PS**				
0-1	234 (98.3)	117 (98.3)	117 (98.3)	
≥2	4 (1.7)	2 (1.7)	2 (1.7)	1.0
**Tumor Biology**				
HR+	190 (79.8)	95 (79.8)	95 (79.8)	
TNBC	48 (20.2)	24 (20.2)	24 (20.2)	1.0
**N. of previous Tx lines**				
≤1	214 (89.9)	104 (87.4)	110 (92.4)	
>1	24 (10.1)	15 (12.6)	9 (7.6)	0.28
**N. of metastatic sites**				
≤1	70 (29.4)	35 (29.4)	35 (29.4)	
>1	168 (70.6)	84 (70.6)	84 (70.6)	1.0
**Visceral disease**				
Yes	153 (64.3)	78 (65.5)	75 (63.0)	
No	85 (35.7)	41 (34.5)	44 (37.0)	0.79
**Time to Metastases**				
Metachronous	203 (85.3)	101 (84.9)	102 (85.7)	
Synchronous	35 (14.7)	18 (15.1)	17 (14.3)	1.0
**Previous Anthracycline Treatment**				
Yes	186 (78.2)	83 (69.7)	103 (86.6)	
No	52 (21.8)	36 (30.3)	16 (13.4)	**0.003**
**Previous Taxane Treatment**				
Yes	183 (76.9)	81 (68.1)	102 (85.7)	
No	55 (23.1)	38 (31.9)	17 (14.3)	**0.002**

Data are presented as n (%) except where otherwise noted. The p value of the χ^2 test assessing the association between each characteristic and the type of treatment received is indicated in the right column of the table. The p value of the test is indicated in bold numbers when statistically significant. *Abbreviations:* C: capecitabine; CV: capecitabine and vinorelbine combination; ECOG PS: Eastern Cooperative Oncology Group performance status; HR: hormone receptor; TNBC: triple negative breast cancer; Tx: Treatment.

Table S2. Clinical and Tumor Characteristics of patients matched on the basis of covariates unequally distributed among treatment groups.

Characteristic	Overall *n* = 176	C subgroup *n* = 88	CV subgroup *n* = 88	*p*
**Age, Years**				
≥65	92 (52.3)	46 (52.3)	46 (52.3)	
<65	84 (47.7)	42 (47.7)	42 (47.7)	1.0
**ECOG PS**				
0-1	170 (96.6)	84 (95.5)	86 (97.7)	
≥2	6 (3.4)	4 (4.5)	2 (2.3)	0.69
**Tumor Biology**				
HR+	137 (77.8)	65 (73.9)	72 (81.8)	
TNBC	39 (22.2)	23 (26.1)	16 (18.2)	0.28
**N. of previous Tx lines**				
≤1	154 (87.5)	73 (83.0)	81 (92.0)	
>1	22 (12.5)	15 (17.0)	7 (8.0)	0.11
**N. of metastatic sites**				
≤1	51 (29.0)	28 (31.8)	23 (26.1)	
>1	125 (71.0)	60 (68.2)	65 (73.9)	0.51
**Visceral disease**				
Yes	118 (67.0)	57 (64.8)	61 (69.3)	
No	58 (33.0)	31 (35.2)	27 (30.7)	0.63
**Time to Metastases**				
Metachronous	146 (83.0)	73 (83.0)	73 (83.0)	
Synchronous	30 (17.0)	15 (17.0)	15 (17.0)	1.0
**Previous Anthracycline Treatment**				
Yes	146 (83.0)	71 (80.7)	75 (85.2)	
No	30 (17.0)	17 (19.3)	13 (14.8)	0.55
**Previous Taxane Treatment**				
Yes	142 (80.7)	73 (83.0)	69 (78.4)	
No	34 (19.3)	15 (17.0)	19 (21.6)	0.57

Data are presented as *n* (%) except where otherwise noted. The *p* value of the χ^2 test assessing the association between each characteristic and the type of treatment received is indicated in the right column of the table. The *p* value of the test is indicated in bold numbers when statistically significant. Abbreviations: C: capecitabine; CV: capecitabine and vinorelbine combination; ECOG PS: Eastern Cooperative Oncology Group performance status; HR: hormone receptor; TNBC: triple negative breast cancer; Tx: Treatment.

© 2020 by the authors. Licensee MDPI, Basel, Switzerland. This article is an open access article distributed under the terms and conditions of the Creative Commons Attribution (CC BY) license (http://creativecommons.org/licenses/by/4.0/).
